# The sexual and gender minority (LGBTQ+) medical trainee: the journey through medical education

**DOI:** 10.1186/s12909-024-05047-4

**Published:** 2024-01-17

**Authors:** Mauricio Danckers, Jake Nusynowitz, Lily Jamneshan, Richard Shalmiyev, Raiko Diaz, Asa E. Radix

**Affiliations:** 1 Division of Pulmonary and Critical Care Medicine, HCA Florida Aventura Hospital, Aventura, FL USA; 2 Herbert Wertheim College of Medicine, Florida International University, Miami, FL USA; 3 Department of Epidemiology, Columbia University Mailman School of Public Health, New York City, New York USA

**Keywords:** LGBTQ+, Medical education, Mentorship, Undergraduate medical education (UME), Graduate medical education (GME)

## Abstract

In this literature overview, we share with the reader challenges faced by LGBTQ + individuals pursuing medical education, from undergraduate to postgraduate training. The LGBTQ + acronym has evolved to encompass the diverse spectrum of sexual orientation and gender identities. Recently, the term “Sexual and Gender Minority” (SGM) has emerged as an umbrella term to provide consistency in research advancing SGM health. The unique obstacles LGBTQ + trainees encounter are highlighted throughout this article, including external factors influencing career decisions, a lack of LGBTQ + healthcare curricula, discriminatory social interactions, limited mentorship opportunities, and a higher mental health burden. These challenges have the capacity to affect educational experiences, personal well-being, and professional growth. Additionally, we examine the impact of inclusive institutional climates on LGBTQ + trainees’ selection of medical schools and residency programs, as they may prioritize inclusiveness and diversity when making their choice. In postgraduate training, LGBTQ + trainees continue to face challenges, exemplified by disparities in placement rates and discriminatory experiences based on sexual orientation and gender identity. We describe the gap in current research and its long-term impact of these challenges on career paths. Hostile environments persist in certain specialties, and the lack of LGBTQ + mentorship and support can hinder academic pursuits. We shed light on the unique and pervasive challenges faced by LGBTQ + trainees throughout their medical education journey, while emphasizing the need for inclusive policies, support systems, and research to address these challenges. With increasing research and studies, we hope to create a medical workforce and community that better represents the diverse communities it serves.

## Background

**Fig. 1:**
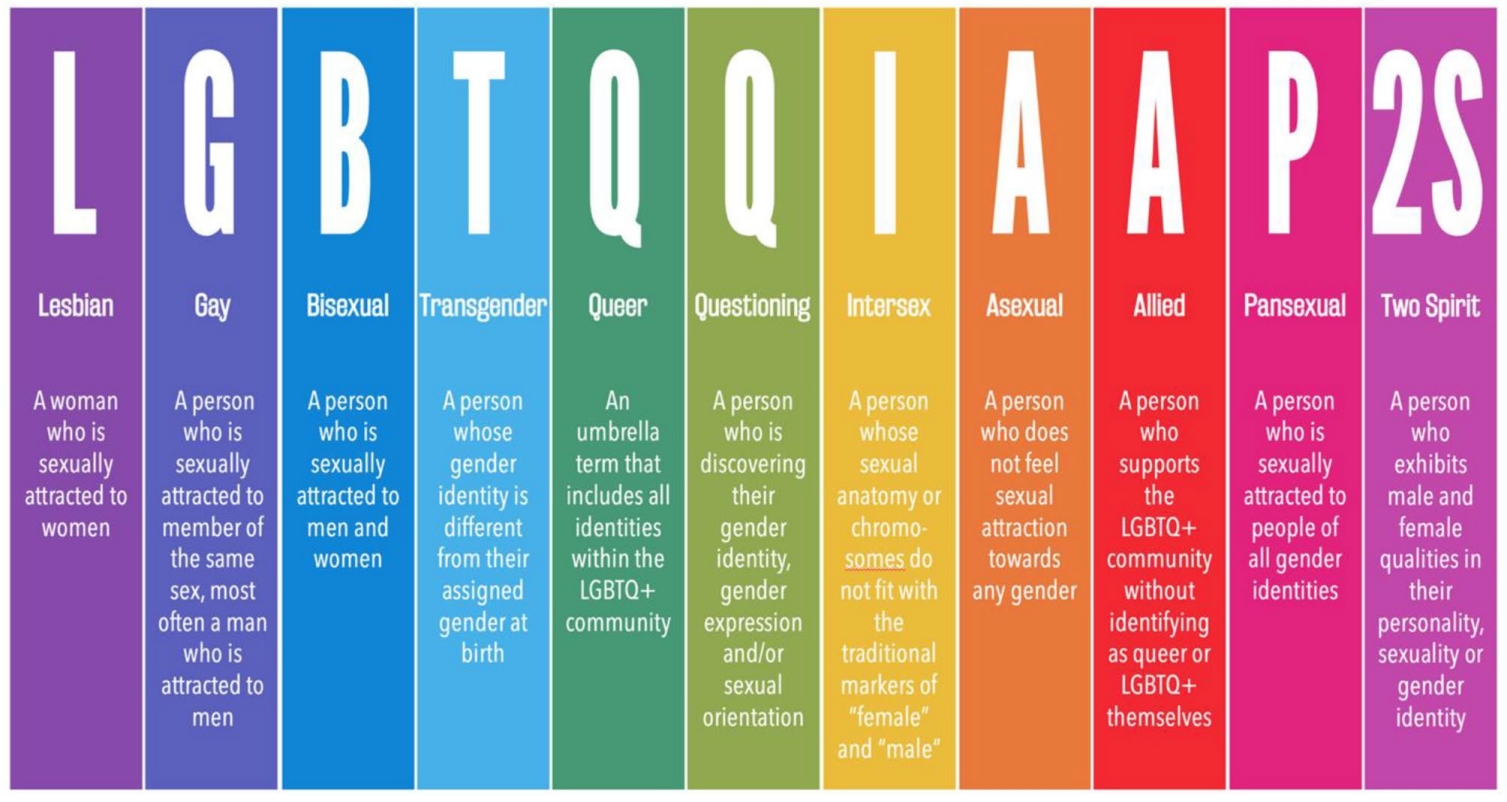
The LGBTQ + acronym.

LGBTQ + is an acronym used to identify the broad community of individuals inclusive of all sexual and gender minorities, as illustrated in Fig. 1. Over decades, this acronym has continuously evolved (LGB, LGBT, LGBTQ, LGBTQ+, LGBTQI+, LGBTQIA+) to fit better the spectrum of sexual orientation and gender identities that fall outside the cisgender, heterosexual, and endosex experience [1].

In 2015, the National Institute of Health, with the establishment of the Sexual and Gender Minority Research Office, adopted the term “Sexual and Gender Minority,” or SGM, as an umbrella term to encompass: (1) those who identify as LGBT, queer, Two-Spirit, Asexual, or intersex (2) those with same-sex or -gender attractions or behaviors, and (3) those with nonbinary constructs of sexual orientation, gender identity or gender expression, or sexual characteristics [2]. This umbrella term was coined to seek consistency in future research to advance SGM health [2].

There is a continuous conversation in the scientific community regarding the most representative terminology. SGM differentiates gender and sexual orientation but is not widely used outside academic and research settings and is less explicit about the specific populations being discussed [3]. More recently, a shift towards using SGD (Sexual and Gender Diversity) has been advocated due to possible negative connotations of the term “minority.”

## Methods

This literature overview surveyed published articles accessing the MEDLINE and MedEDPORTAL databases. Two authors reviewed and appraised the literature focusing on three distinctive domains: (1) pre-medical education of the LGBTQ + individual, (2) undergraduate medical education of the LGBTQ + medical trainee, and (3) graduate medical education of the LGBTQ + medical trainee. The findings were synthesized following a thematic analysis and reported narratively. We used the terms SGM and the acronym LGBTQ + in this manuscript in correlation with their use in the medical literature and the limitation of each terminology’s context. The aim of our literature overview is to provide our readers with a deeper understanding of LGBTQ + individuals’ challenges through medical training and the status of LGBTQ + trainees in medical education.

### Advocacy for LGBTQ + trainees in medical education

Although significant strides have been made in the advocacy of the LGBTQ + community, discrimination, and marginalization continue to be tangible at different levels of societal interaction. In healthcare, efforts are being made to create a medical workforce that more accurately mirrors the communities they serve. The shift aims to overcome minority underrepresentation in undergraduate medical education (UME) and graduate medical education (GME) training programs, to promote educational curricula for the medical care of the LGBTQ + patient, to call for institutional reform to support inclusive recruitment and work environments, and for the equitable professional advancement of all trainees in the medical field.

The LGBTQ + trainee faces unique challenges throughout medical education that could lead to disadvantageous training experiences and an isolated environment for formative learning. Among those: (1) external factors affecting their decision to join a medical school, their selection of specialization field, and career path [4–11], (2) lack of LGBTQ + health care educational curricula perpetuating the sense of invisibility in medical education [12–15], (3) discriminatory social interactions with peers and supervisors in training environment [4–6, 8, 16–19, 24−27], (4) limited professional mentorship opportunities and depleted opportunities for professional advancement [4–7, 10, 28–30, 31], (5) complex interactions with patients and their cultural biases [25, 26], and (6) higher mental health burden [5, 16, 18, 19, 25, 32, 33]. Understanding the complex journey of LGBTQ + trainees will provide medical educators with the skill sets to propel cultural change in their training that starts at the bedside and ends with adopting more significant institutional and national reforms.

Despite the many difficulties LGBTQ + trainees experience during their UME and GME, evidence-based guidance to identify and overcome said challenges is lacking. This gap is accentuated as the LGBTQ + trainee moves further into GME.

### The LGBTQ + trainee journey

LGTBQ + trainees experience challenges related to their sexuality/and or gender identity early in their formative years, long before applying to medical school. LGBTQ + youth are more likely to be bullied and face sexual violence than their cisgender, heterosexual peers, resulting in higher school dropout rates and limitation of educational opportunities [17]. Parental rejection can lead to homelessness and further curtail educational opportunities [18]. Many LGBTQ + individuals start college with unique personal challenges related to identity development, disclosure (“coming out”), establishing same-sex romantic relationships, and overcoming internalized stigma while experiencing harassment, violence, and discrimination [19]. Therefore, resilience and personal survivorship have been a significant facet of the LGTBQ + individual’s experience by the time they achieve a place in medical education.

“Coming out” describes the process of disclosing one’s sexual and gender identity to others and has been described as one of the most stressful yet pivotal experiences that an LGBTQ + person faces in their lifetime [20]. Contrary to common belief, “coming out” is not a one-time occurrence but rather a continuous event throughout one’s life, such as when joining a medical school and throughout medical training and practice. The stressors that accompany the experience of coming out can be attributed to enacted and anticipated stigma. Enacted stigma relates to current and ongoing discrimination and harassment from external sources, such as at home, in the workplace, or within their community. Anticipated stigma includes the expectation of adverse events, such as a lack of acceptance and ostracism from family, friends, colleagues, and society [21]. LGBTQ + students in science, technology, engineering, and math (STEM) are more likely to experience career limitations, harassment, and professional devaluation than their non-LGBTQ + peers [3]. When compounding these psychosocial stressors with the remarkable intensity of medical training, the potential for negative mental, emotional, and physical consequences on the LGBTQ + student is immense.

**Fig. 2:**
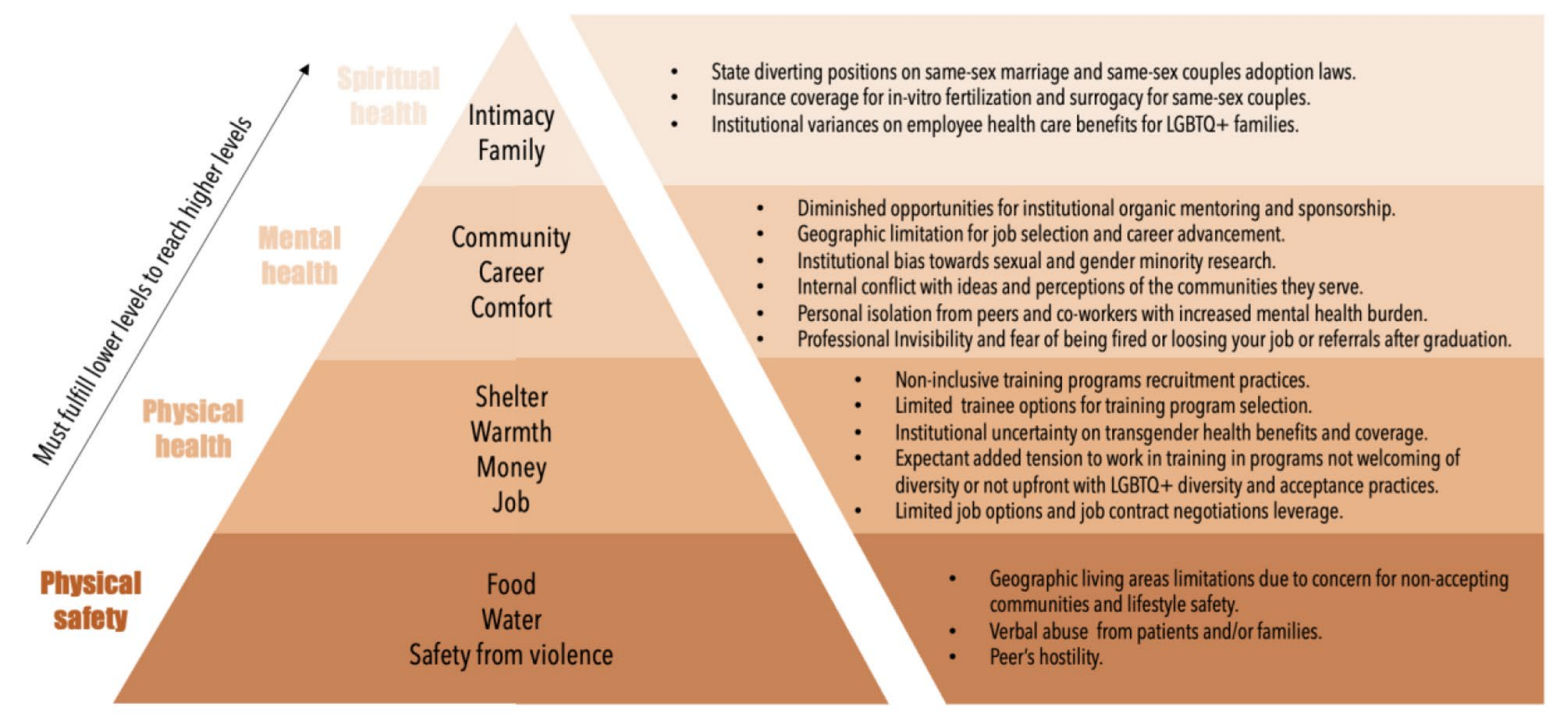
LGBTQ + medical trainee barriers in Maslow’s hierarchy of human need fulfillment ^a^. ^a^ Modified from O’Hanlan et al. A review of medical consequences of homophobia and suggestions for resolution. JGLMA 1997:1:25–39

The professional development of the LGBTQ + individual could be hindered by factors, founded on discrimination, that challenge human need fulfillment [22, 23]. We have adapted prior published work [22] and propose a specific set of factors that directly impact the professional success of the LGBTQ + medical trainee following the Maslow’s Hierarchy of human need fulfillment theoretical framework in Fig. 2 [23]. The persistence of oftentimes hostile environments makes the adjustment to the increasing rigor of healthcare disproportionately more difficult for those in the LGBTQ + community. It leads to poorer outcomes, such as higher levels of burnout [24], less job satisfaction, and negative mental and physical consequences [25]. Lesbian, gay, or bisexual students mistreated for their sexual orientation had an 8-fold higher predicted probability of burnout compared with heterosexual students [24], with a higher mistreatment rate across multiple categories (humiliation, not specific to identity, specific to gender, race/ethnicity, and sexual orientation).

Factors such as geography, finances, and lifestyle come into play when considering the process of pursuing medical school. For many LGBTQ + trainees, finding a welcoming and diverse school is crucial to their application. Demographic data collection of SGM students from medical school admission and enrollment processes is undervalued [34]. Certain institutional climates can be perceived as non-inclusive and unwelcoming to minorities. Even when institutional policies are considerate of sexual orientation and gender diversity, institutions must play an active role in recruitment that emphasizes inclusive practices during recruitment and equity during medical training.

A study among graduating medical students reported SGM students having a higher proportion of mistreatment (43.5% vs. 23.6%) and discrimination based on sexual orientation (32.1% vs. 1.0%) than their heterosexual counterparts [24]. The study did not survey for discrimination rate differences between cis- and transgender students. Higher levels of depression, lower levels of perceived social support, and more discomfort with disclosure of sexual orientation have been reported in LGBTQ + medical students compared to non-LGBTQ + students, with most of their campuses described as non-inclusive [16]. Another study showed higher rates of bullying by other students (20.0% vs. 13.9%) and suicide contemplation (14.8% vs. 8.8%) compared to non-LGBTQ + students. Moreover, surgical specialties were perceived as having the lowest acceptance of LGBTQ + trainees [5].

It is also important to consider intersectionality, i.e., the overlapping and interdependent systems of oppression, that can impact LGBTQ + trainees and professionals who have additional marginalized identities, including their race, ethnicity, ability, and immigrant status, among others. For example, a trainee who is an African American transgender woman may experience unique challenges due to the complex, cumulative, and intersecting effects of racism, transphobia, and misogyny. Although there is limited research on intersectionality and LGBTQ + trainees, this phenomenon has been well described among other individuals holding multiple marginalized identities, including African American women physicians who experience isolation and self-doubt because of gender- and race-based macro and microaggressions [35].

Due to such conditions, LGBTQ + trainees may be more limited in personal and professional growth opportunities. Many states have become increasingly outspoken in supporting anti-LGBTQ + laws in recent years. Florida’s “Don’t Say Gay’’ bill, initially applied to grades K-3, has since been extended to ban classroom discussion on sexual orientation and gender identity up to the 12th grade [36]. This expansion has raised concerns due to its implications that threaten to worsen an existing hostile school climate for LGBTQ + youth. Notably, 52% of LGBTQ + students have considered dropping out of school due to hostility, and 72% report having no LGBTQ + topics taught in any classes [37]. These restrictions send a discriminatory message that being LGBTQ + is wrong and stigmatizes both LGBTQ + youth and the community at large.

This critical change and other decisive attacks on the LGBTQ + community, such as SB 1438 and its attempted “drag ban” and Supreme Court decisions limiting affirmative action in higher education and LGBTQ + protections, may play a role in the medical school selection process for impacted applicants. LGBTQ + students in states like Florida may choose between pursuing medical education in a traditionally more “accepting” location and saving money with in-state tuition costs. Further research is needed to evaluate the long-term impact of mentorship absenteeism, unwelcoming training, and communities’ environments affecting LGBTQ + medical students on their career path, professional achievements, and community service.

### Postgraduate training experience for the LGBTQ + trainee

While most existing literature regarding LGBTQ + trainees relates to student accomplishments in UME, research in GME is limited. The call for further exploration into the impact of GME on the LTBTQ + trainee is crucial, with research needs spanning all residency specialties and more accentuated during fellowship training. So far, studies in post-graduate training have suggested that the LGBTQ + trainee’s challenges may be more prominent during residency [6]. A retrospective cohort of residency applicants found a significant disadvantage in underrepresented minorities, with the highest rate of unsuccessful GME placement, and called for equity metrics in residency spot allocation [7]. Although this cohort did not differentiate minority groups, we suspect the findings for SGM trainees would be similar, if not more discouraging.

A large cohort study addressing disparities in medical students’ placement rates into graduate medical programs pointed out lower rates for female and underrepresented minority students [7]. Although inequalities in the residency selection process continue to be encountered, a gender binary limiting option within the survey did not allow for further assessment of the placement of non-binary and non-conforming gender medical students and their inherent challenges. Consensus reports call for inclusiveness in these research tools to more thoroughly capture the spectrum of sexual and gender-diverse groups to mitigate underreporting and avoid perpetuating their marginalization in medical education research [3].

When selecting a residency program, the LGBTQ + trainee often prioritizes the inclusiveness and diversity of the community in which the training program is located. In a study of emergency medicine residency applicants, LGBTQ + applicants ranked the ability to live in a particular setting (urban, suburban, and rural), neighborhood and community, and patient population as having greater average importance when compared with non-LGBTQ + applicants [8]. Transgender and non-binary residents or recent graduates of a US residency program were surveyed on their residency interview process, revealing that a high percentage of applicants felt unsafe to disclose or discuss their gender identity (69.2%), were misnamed or misgendered through incorrect pronouns during their interview (42.3%) and thought they were ranked lower than their qualifications due to their gender identity (26.9%) [9].

Residency training continues to be disadvantageous for educating LGBTQ + trainees, specifically transgender and non-binary residents [26]. Alarmingly, 85% of transgender and non-binary residents self-reported experiencing microaggressions, while nearly one-quarter of transfeminine and non-binary trainees reported macroaggressions, mostly from program faculty [26]. A survey conducted among gender non-binary and transgender physicians and medical students revealed that most respondents had not disclosed their identity with their medical school or residency program. Respondents also reported that barriers based on gender identity/expression are more prevalent in residency [6]. Most individuals reported censoring speech or mannerisms to avoid unintentional disclosure of their gender identity and detail hearing derogatory terms referring to transgender and nonbinary individuals [6].

The often-hostile training environments reported during GME are consistent with a perpetuation of practices seen in UME and recruitment seasons. These inequitable environments require a heightened amount of resilience and grit by the SGM individual to attain the same level of success. As a result, these efforts can be misplaced as tools that amplify structural inequity and injustice rather than to promote success more broadly [38].

In a large-scale survey looking at mistreatment in the workplace among emergency medicine residents, including 483 residents self-identified as LGBTQ + trainees, discrimination based on sexual orientation or gender identity was reported in 3.1% of all residents and 26.9% of LGBTQ + residents [9, 27]. The same study reported that most LGBTQ + trainees who reported discrimination identified patients and/or their families as the primary source of discrimination (56.2%), followed by other residents (13.8%) and attending physicians (11.5%) [27]. An adjusted model showed that LGBTQ + trainees were two times more likely to have suicidal thoughts than their non-LGBTQ + counterparts [27]. A quality study with significant medical trainee representation reported biases ranging from patient refusal of care, to explicitly racist, sexist, or homophobic remarks and belittling compliments or jokes. In this study, targeted physicians reported emotional responses such as exhaustion, self-doubt, and cynicism, while non-targeted bystanders expressed moral distress and uncertainty about how to respond [28].

Social factors also play a significant role in the choice of residency and fellowship for the LGBTQ + trainee, yet little is known about their selection process and determinants. AAMC US Physician Workforce data from 2019 showed that racial minorities are vastly underrepresented in medicine, with surgery failing the most to address this social disparity [39]. The surgical field has traditionally been perceived as a non-diverse training field with a predominantly white heterosexual cis-male trainee population [5] that lacks diversity. This environment, described by some medical trainees as a “boys club” or “fraternity” [5], could intentionally or not, unwelcome LGBTQ + trainees. A 2022 survey completed by almost 6,000 residents showed that SGM trainees, specifically general surgery, represent approximately 5% of the total resident body but report a statistically significant difference in harassment, mistreatment, bullying, discrimination, homophobic remarks, and suicidality, primarily from attending physicians [40]. These results are consistent with previous studies and reviews [29, 39, 41].

LGBTQ + trainees might shy away from surgical fields due to scarce LGBTQ + faculty and mentoring. Trainees may doubt fair academic promotion and support for research opportunities in LGBTQ + health. Trainees may also experience fear of reprisal, worry of increased animosity, the belief that nothing would be done, and lack of safety of support [29, 40]. This could be substantial in specialties and subspecialties that do not challenge the assumption of trainee homogeneity and perpetuate a trainee stereotype apt for professional success.

Other prestigious specialties, determined by an objective index that included the number of available positions and median income, have also been reported as less SGM inclusive [10]. Studies have shown that SGM trainees perceive certain specialties as more inclusive (psychiatry, family medicine, pediatrics, preventative medicine, and internal medicine) and others as less inclusive (orthopedics, neurosurgery, thoracic surgery, general surgery, and colorectal surgery) [10]. Sex and gender identity strongly influence LGBTQ + trainees’ specialty of choice, along with other determinants like personality fit, specialty content, and work-life balance [10].

Internal medicine subspecialty fellowship pipelines, such as pulmonary and critical care medicine, have plateaued in terms of gender diversity from 2009 to 2018 and have worsened for racial and ethnic groups trainee representation [11]. A specific pipeline for LGBTQ + trainees remains unknown, and the factors impacting its course are understudied.

During their residency and fellowship training, LGBTQ + trainees report high interest in pursuing careers in academia. In a survey of 54 LGBTQ + trainees and health care providers, 81.1% of trained physicians were interested in academia [4]. LGBTQ + trainees’ interests were positively impacted by their desire to help others succeed, teaching, the competitive nature of the position, compatibility with personality and interest, and the mentor/role model influence [4]. The poor recognition of LGBTQ + scholarship, lack of LGBTQ + mentoring and networking opportunities, and hostile institutional climates were barriers to pursuing an academic career [4]. One in five trainees reported that their academic health centers did not provide a supportive environment for LGBTQ+-related research and educational activities or engage in service or community activities in LGBTQ + care [4].

Highly qualified LGBTQ + providers completing their training may select urban over rural areas to seek well-established LGBTQ + communities in cities perceived as friendly for personal and professional development. This geographic limitation may impair the LGBTQ + trainee in selecting a highly desirable job, as they seek positions not based on their qualifications but on social adaptation and future well-being.

Faculty may be unaware of crucial social aspects and fail to meet the needs of LGBTQ + patients [42, 43]. Furthermore, there is a high likelihood that faculty and training peers might have never knowingly interacted with LGBTQ + individuals, perpetuating bias towards LGBTQ + trainees. The amount of contact with LGBTQ + faculty, residents, students, and patients, and the perceived quality of that contact, has been associated with reduced explicit bias in medical training [44].

In the search for social change in GME, most training programs’ curricula have started to emphasize binary gender equity and racial minority diversity and inclusion. The inclusion of SGM groups may carry less emphasis in these initiatives. Equality for the LGBTQ + community is expanding rapidly, and the healthcare system and medical education should serve as an example of that expansion.

To begin to address these inadequacies, evidence-based strategies must be implemented. Successful higher-level interventions aimed to target LGBTQ + health curricula include diverse instructional methods such as lecture-based didactics, online modules, and simulations. Longitudinal curricula, like the LGBTQ Health Pathway, have been effective within UME, incorporating preclinical and clinical components such as online modules, didactic courses, longitudinal community service/advocacy work, a scholarly project, and a clinical clerkship in LGBTQ + health [45]. Pathways such as this could theoretically be molded for GME and specialty-specific training.

Furthermore, at the individual level, mentorship can play a crucial role, with LGBTQ + mentors positively influencing trainees’ confidence, professional success, and sense of belonging. Mentorship and sponsorship, whether through established processes or organic trainee-faculty interactions, can foster a sense of belonging while cultivating the professional development of both the mentee and mentor [46]. The evidence-based strategies presented here only scratch the surface, and comprehensive changes must occur at multiple levels (individual, program-level, institutional, societal, etc.)

## Conclusion

In conclusion, LGBTQ + trainees face unique medical education challenges extending from undergraduate medical education to postgraduate training and beyond. The SGM trainee’s journey toward acceptance and self-discovery requires exceptional resiliency and survivorship. Challenges encountered during UME include external factors, such as financial limitations, lack of representative curriculum, inflammatory social interactions, inadequate mentorship opportunities, discriminatory interactions with patients, and a significant mental health burden. Furthermore, harsh training environments and discriminatory practices in GME tend to perpetuate these inequities. Despite the growing body of research, evidence-based guidance to overcome these challenges is still lacking, particularly when considering GME. Medical educators and administrators must work toward understanding the complex journey of LGBTQ + trainees and provide them with the necessary skill sets to succeed and propel meaningful cultural change. This essential shift toward inclusivity can potentially create a medical workforce that more accurately mirrors the colorful community it serves.

## Data Availability

Not applicable.

## References

[1] Blakemore E. From LGBT to LGBTQIA+: the evolving recognition of identity. October; 2021.

[2] National Academies of Sciences, Engineering, and, Medicine. NIH. FY 2016–2020 Strategic Plan to Advance Research on the Health and Well-being of Sexual and Gender Minorities. 2015.

[3] National Academies of. Sciences, Engineering, and Medicine. Measuring Sex, Gender Identity, and Sexual Orientation. 2022. 35286054

[4] Sánchez NF, Rankin S, Callahan E, Ng H, Holaday L, McIntosh K (2015). LGBT Trainee and Health Professional perspectives on Academic Careers–facilitators and challenges. LGBT Health.

[5] Madrigal J, Rudasill S, Tran Z, Bergman J, Benharash P (2021). Sexual and gender minority identity in undergraduate medical education: impact on experience and career trajectory. PLoS ONE.

[6] Dimant OE, Cook TE, Greene RE, Radix AE (2019). Experiences of transgender and gender Nonbinary Medical Students and Physicians. Transgend Health.

[7] Nguyen M, Chaudhry SI, Desai MM, Hajduk AM, McDade WA, Fancher TL (2022). Rates of medical student placement into graduate medical education by sex, race and ethnicity, and socioeconomic status, 2018–2021. JAMA Netw Open.

[8] Weygandt PL, Smylie L, Ordonez E, Jordan J, Chung AS (2021). Factors influencing emergency medicine residency choice: diversity, community, and recruitment red flags. AEM Educ Train.

[9] Kvach EJ, Weinand J, O’Connell R (2021). Experiences of transgender and nonbinary physicians during medical residency program application. J Graduate Med Educ.

[10] Sitkin NA, Pachankis JE (2016). Specialty choice among sexual and gender minorities in medicine: the role of specialty prestige, perceived inclusion, and medical school climate. LGBT Health.

[11] Santhosh L, Babik JM (2020). Diversity in the pulmonary and critical Care Medicine Pipeline. Trends in gender, race, and ethnicity among applicants and fellows. ATS Sch.

[12] Hayes V, Blondeau W, Bing-You RG (2015). Assessment of medical student and resident/fellow knowledge, comfort, and training with sexual history taking in LGBTQ patients. Fam Med.

[13] Dubin SN, Nolan IT, Streed CG Jr, Greene RE, Radix AE, Morrison SD. Transgender health care: improving medical students’ and residents’ training and awareness. Advances in medical education and practice. 2018:377– 91. 10.2147/AMEP.S147183 PMC5967378 29849472

[14] Honigberg MC, Eshel N, Luskin MR, Shaykevich S, Lipsitz SR, Katz JT (2017). Curricular time, patient exposure, and comfort caring for lesbian, gay, bisexual, and transgender patients among recent medical graduates. LGBT Health.

[15] Pregnall AM, Churchwell AL, Ehrenfeld JM (2021). A call for LGBTQ content in graduate medical education program requirements. Acad Med.

[16] Lapinski J, Sexton P (2014). Still in the closet: the invisible minority in medical education. BMC Med Educ.

[17] Clayton HB, Kilmer G, DeGue S, Estefan LF, Le VD, Suarez NA (2023). Dating violence, sexual violence, and bullying victimization among High School Students -Youth Risk Behavior Survey, United States, 2021. MMWR Suppl.

[18] Rhoades H, Rusow JA, Bond D, Lanteigne A, Fulginiti A, Goldbach JT (2018). Homelessness, Mental Health and Suicidality among LGBTQ Youth Accessing Crisis services. Child Psychiatry Hum Dev.

[19] Sanlo R (2004). Lesbian, gay, and bisexual college students: risk, resiliency, and retention. J Coll Student Retention: Res Theory Pract.

[20] VC. Homosexual identity formation: a theoretical model. J Homosex. 1979 Spring;4(3):219–35. 10.1300/J082v04n03_01. 10.1300/J082v04n03_01 264126

[21] Rosati F, Pistella J, Nappa MR, Baiocco R (2020). The coming-out process in family, social, and religious contexts among young, middle, and older Italian LGBQ + adults. Front Psychol.

[22] O’Hanlan KA, et al. A review of the Medical consequences of Homophobia with suggestions for Resolution. J Gay Lesbian Med Association. Mar. 1997;1(1):25–39. 10.1023/b:jola.0000007009.83600.ae.

[23] Maslow AH (1943). A theory of human motivation. Psychol Rev.

[24] Hill KA, Samuels EA, Gross CP, Desai MM, Sitkin Zelin N, Latimore D (2020). Assessment of the prevalence of Medical Student Mistreatment by Sex, Race/Ethnicity, and sexual orientation. JAMA Intern Med.

[25] Eliason MJ, Streed C, Henne M (2018). Coping with stress as an LGBTQ + Health Care Professional. J Homosex.

[26] Weinand JD, Kvach EJ, O’Connell R. Experiences of transgender and nonbinary physicians during residency training. Int J Transgender Health. 2022:1–11. 10.1080/26895269.2022.2098219 PMC10601501 37901054

[27] Lall MD, Bilimoria KY, Lu DW, Zhan T, Barton MA, Hu Y-Y (2021). Prevalence of discrimination, abuse, and harassment in emergency medicine residency training in the US. JAMA Netw open.

[28] Wheeler M, de Bourmont S, Paul-Emile K, Pfeffinger A, McMullen A, Critchfield JM (2019). Physician and trainee experiences with patient bias. JAMA Intern Med.

[29] Lee KP, Kelz RR, Dubé B, Morris JB (2014). Attitude and perceptions of the other underrepresented minority in surgery. J Surg Educ.

[30] Field S, Rajewski A (2021). Challenges facing LGBTQ + early-career scientists and how to engage in changing the status quo. Plant Cell.

[31] Wittlin NM, Dovidio JF, Burke SE, Przedworski JM, Herrin J, Dyrbye L, et al. Contact and role modeling predict bias against lesbian and gay individuals among early-career physicians: a longitudinal study. Soc Sci Med. 2019;238:112422. 10.1016/j.socscimed.2019.112422 PMC6744977 31391147

[32] Weiss J, Balasuriya L, Cramer LD, Nunez-Smith M, Genao I, Gonzalez-Colaso R (2021). Medical students’ demographic characteristics and their perceptions of faculty role modeling of respect for diversity. JAMA Netw open.

[33] Samuels EA, Boatright DH, Wong AH, Cramer LD, Desai MM, Solotke MT, et al. Association between sexual orientation, mistreatment, and burnout among US medical students. JAMA Netw open. 2021;4(2):e2036136–e. 10.1001/jamanetworkopen.2020.36136 PMC7856540 33528552

[34] Gamble RM, Pregnall AM, Deng A, Ehrenfeld JM, Talley J (2021). US medical school admissions and enrollment practices: status of LGBTQ inclusivity. J Osteopath Med.

[35] Chilakala A, Camacho-Rivera M, Frye V (2022). Experiences of race- and gender-based discrimination among black female physicians. J Natl Med Assoc.

[36] Woo A, Diliberti MK. How Florida’s Expansion of Dont Say Gay Law Will Hurt Students and Teachers Across the United States. 2023.

[37] Kosciw J, Clark C, Menard L. 2021 National School Climate Survey. 2023.

[38] Montgomery BL (2020). Academic leadership: gatekeeping or groundskeeping?. J Values-Based Leadersh.

[39] Clarke CN (2022). More than a moment: why surgical societies must continuously strive for diversification and creating a culture of equity and inclusivity in academic surgery. Am J Surg.

[40] Heiderscheit EA, Schlick CJR, Ellis RJ (2022). Experiences of LGBTQ + residents in US general surgery Training Programs. JAMA Surg.

[41] Eliason MJ D, SL (2011). Lesbian, gay, bisexual, and transgender (LGBT) physicians’ experiences in the workplace. J Homosex.

[42] Beagan B, Fredericks E, Bryson M (2015). Family physician perceptions of working with LGBTQ patients: physician training needs. Can Med Educ J.

[43] Berner AM, Hughes DJ, Tharmalingam H, Baker T, Heyworth B, Banerjee S (2020). An evaluation of self-perceived knowledge, attitudes and behaviours of UK oncologists about LGBTQ + patients with cancer. ESMO Open.

[44] Phelan SM, Burke SE, Hardeman RR, White RO, Przedworski J, Dovidio JF (2017). Medical School Factors Associated with changes in Implicit and Explicit Bias against Gay and Lesbian people among 3492 Graduating Medical Students. J Gen Intern Med.

[45] Gibson AW, Gobillot TA, Wang K (2020). A novel curriculum for medical student training in LGBTQ healthcare: a regional pathway experience. J Med Educ Curric Dev.

[46] Beanlands RA, Robinson LJ, Venance SL (2020). An LGBTQ + mentorship program enriched the experience of medical students and physician mentors. Can Med Educ J Dec.

